# Different roles of integrin-β_1_ and integrin-α_v_ for type IV secretion of CagA versus cell elongation phenotype and cell lifting by *Helicobacter pylori*

**DOI:** 10.1371/journal.ppat.1008135

**Published:** 2020-07-21

**Authors:** Nicole Tegtmeyer, Steffen Backert

**Affiliations:** Department of Biology, Division of Microbiology, Friedrich Alexander University Erlangen-Nuremberg, Erlangen, Germany; University of Illinois, UNITED STATES

**Formal comment for Zhao et al. 2018** (PLoS Pathog. 14: e1007359).

Using CRISPR/Cas9 gene knockout, Zhao *et al*. [1] unexpectedly reported that neither heterodimers of integrin-β_1_ nor integrin-α_v_ are essential for translocation of CagA by a type IV secretion system (T4SS) and for induction of the cell elongation phenotype by *H*. *pylori*. We performed infection experiments using the reported AGS wild-type and isogenic knockout cells for integrin-β_1_ (ΔITGB1) and integrin-α_v_/integrin-β_4_ (ΔITGAvB4) [1] with various worldwide T4SS-positive strains including TN2-GF4, G27 and P12 used by the authors. The absence or presence of integrin-β_1_ and integrin-α_v_ in the respective cell lines was confirmed (Fig 1A–1C). Subsequently, the blots were probed with α‐CagA and α‐PY‐99 antibodies to monitor CagA phosphorylation (CagA^PY^), indicating successful translocation. Infection with all strains produced strong CagA^PY^ signals in all cell lines. Densitometric quantification revealed some differences among band intensities between cell lines and strains, but did not reach statistical significance (Fig 1A–1C). Thus, we can confirm that the knockout of ITGB1 or ITGAvB4 in AGS cells did not significantly affect CagA delivery and phosphorylation. Surprisingly, however, we found that knockout of ITGB1 completely abolished the cell elongation phenotype (Fig 1D and 1E), while inactivation of ITGAvB4 even enhanced this phenotype significantly (Fig 1F). These data contradict above findings, suggesting that corresponding pictures have been differently interpreted [1]. Our data instead suggest a yet unrecognized and important role of ITGB1 and suppressive function for ITGAvB4. However, numerous pressing questions arose by the above new findings.

**Fig 1:**
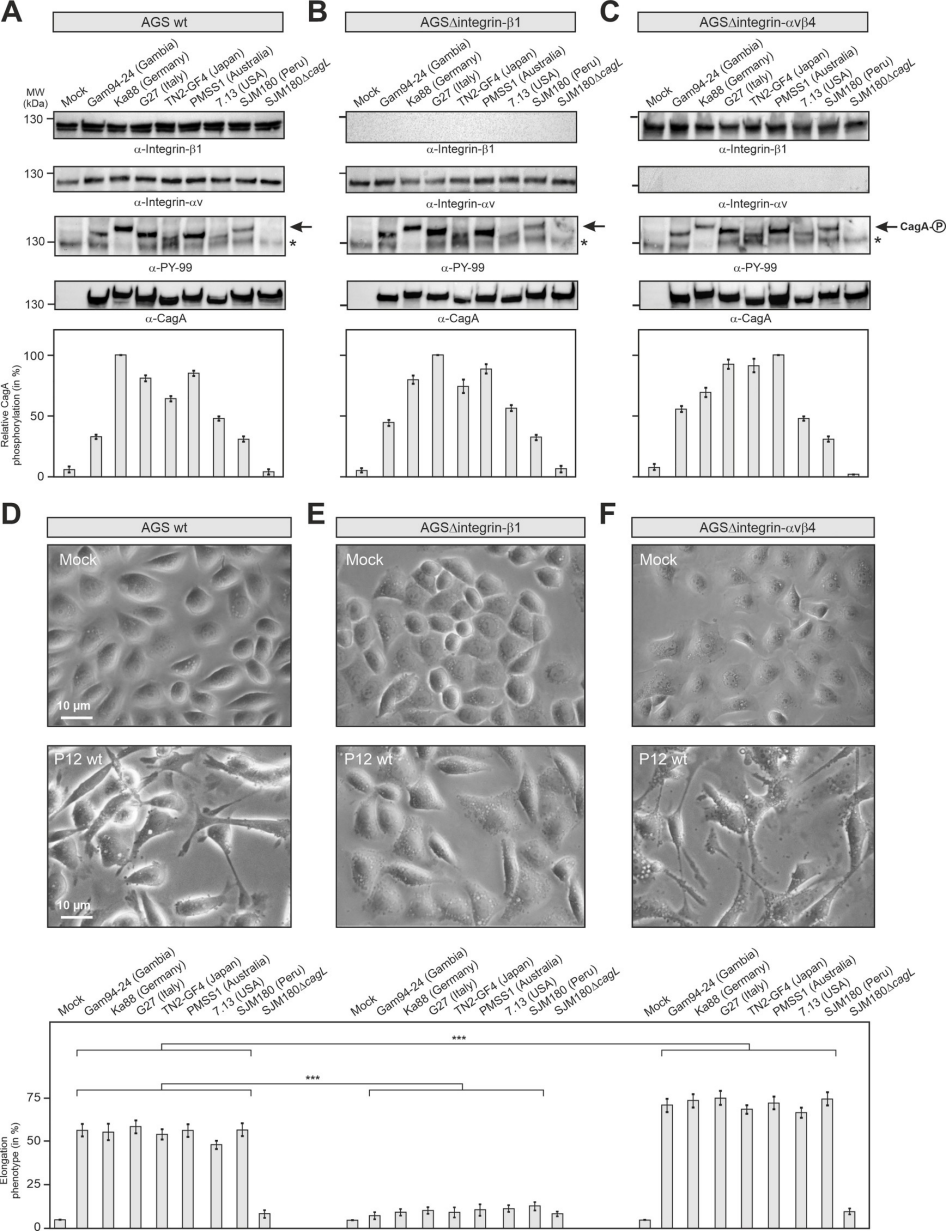
Quantification of CagA phosphorylation and elongation phenotype upon *H*. *pylori* infection of AGS gastric epithelial cell types. (A) AGS wild-type, (B) AGSΔITGB1 and (C) AGSΔITGAvB4 knockout cells [1] were infected with the indicated *H*. *pylori* strains for 6 hours using a multiplicity of infection of 50. Western blotting using specific antibodies confirmed the integrin-β_1_ and integrin-α_v_ expression status (top panels). Probing with α‐CagA and α‐PY‐99 antibodies (middle panels) was applied to quantitate the degrees of CagA phosphorylation in triplicates by densitometry (bottom panels). The asterisks indicate the position of a phosphorylated ~125 kDa host cell protein on the gels. (D-F) Phase contrast microscopy of non-infected vs. infected AGS cell lines (top and middle panels). The elongation phenotype was quantitated in triplicate experiments showing inhibition in infected ΔITGB1 cells and enhancement in ΔITGAvB4 cells compared to the AGS wild-type control (bottom panels).

First, how can one explain the negative results concerning the role of integrins on T4SS-dependent CagA delivery? AGS cells express a series of integrin-β_1_-based heterodimers (α_1_β_1_, α_2_β_1_, α_3_β_1_, α_5_β_1_, α_6_β_1_ and α_9_β_1_), integrin-α_v_ heterodimers (α_v_β_5_ and α_v_β_6_) and integrin-α_6_β_4_ [1,2]. Knockout of the β_1_ gene should therefore abrogate surface exposure of every possible β_1_-comprising integrin heterodimers [1]. To avoid functional substitution of β_1_ by other integrin combinations (such as α_v_β_5_, α_v_β_6_ or α_6_β_4_), integrins α_v_ and β_4_ were also inactivated, and did not significantly compromise CagA translocation either [1]. These data contradict earlier publications that CagA delivery was achieved in integrin-β_1_ expressing GE11 or GD25 mouse cell lines, but not in their isogenic integrin-β_1_ knockouts [2,3]. However, bacterial binding to these cells may differ, but was not tested. In addition, we know today that these cells do not express human CEACAMs, which are newly discovered receptors for CagA delivery [4,5]. It was proposed that CagA translocation in the integrin-β_1_-expressing counterparts was only at low background levels in cells lacking human CEACAMs with integrins having only a small supportive effect on CagA delivery [1]. Other earlier data showed that two function-interfering integrin-β_1_ antibodies or recombinant CagA fragments, comprising the integrin-β_1_ binding site, downregulated CagA translocation [2,3,6]. In the light of the new data, these previous observations can be interpreted as indirect impact of integrin-β_1_ on CagA translocation, such as steric hindrance of CEACAM receptors, together implying that integrin-β_1_ is not essential.

Second, how can we explain the different results concerning the role of integrins on the elongation phenotype? CagA translocation by *H*. *pylori* and subsequent phosphorylation were reported to be essential for the elongation phenotype [7,8]. The essentiality of the protein for phenotypical outcome was later confirmed by ectopic expression of CagA in AGS cells [9]. CagA^PY^ induces this phenotype through a cell retraction defect during cell movement (Fig 2A and 2B). Time-lapse video microscopy has shown that infected cells undergo elongation because they failed to release their “back ends” upon cell locomotion [10]. These “back ends” represent enlarged focal adhesions (FAs) and their disassembly is inhibited resulting in elongated cell projections [8]. Thus, it was proposed that the function of CagA^PY^ is to strengthen the FAs in epithelial cells, preventing excessive cell lifting during infection [11]. To achieve this goal, CagA^PY^ manipulates the activities of tyrosine phosphatase SHP2 [9] and actin-binding protein cortactin [11,12], resulting in the deregulated action of focal adhesion kinase FAK [11–13]. In turn, FAK activity controls cell adhesion to the extracellular matrix (ECM) through integrin. Besides FAK, the FA complex comprises many other signaling factors including kinase Src and cytoskeletal proteins talin, paxillin, vinculin, p130Cas and α-actinin, connecting to the actin-cytoskeleton [14–16]. The cytoplasmic domain of integrin-β_1_ was exemplary shown to interact directly with α-actinin, paxillin, talin and FAK [14–16], and is sufficient for its recruitment to preformed FAs and signal transduction to FAK [16–19]. This can explain why integrin-β_1_ is absolutely required for the *H*. *pylori-*induced elongation phenotype in AGS cells. In the absence of integrin-β_1_, α_v_-containing FAs can be formed [20], however, we postulate that cortactin and SHP2 only have a low or no impact on the turnover of FAs upon infection of ΔITGB1 cells or the composition of the FAs and/or phosphorylation status of FA proteins might have changed (Fig 2C). On the other hand, knockout of ITGAvB4 in AGS cells enhances the elongation phenotype by a yet unknown mechanism, which needs to be elucidated in future studies.

**Fig 2:**
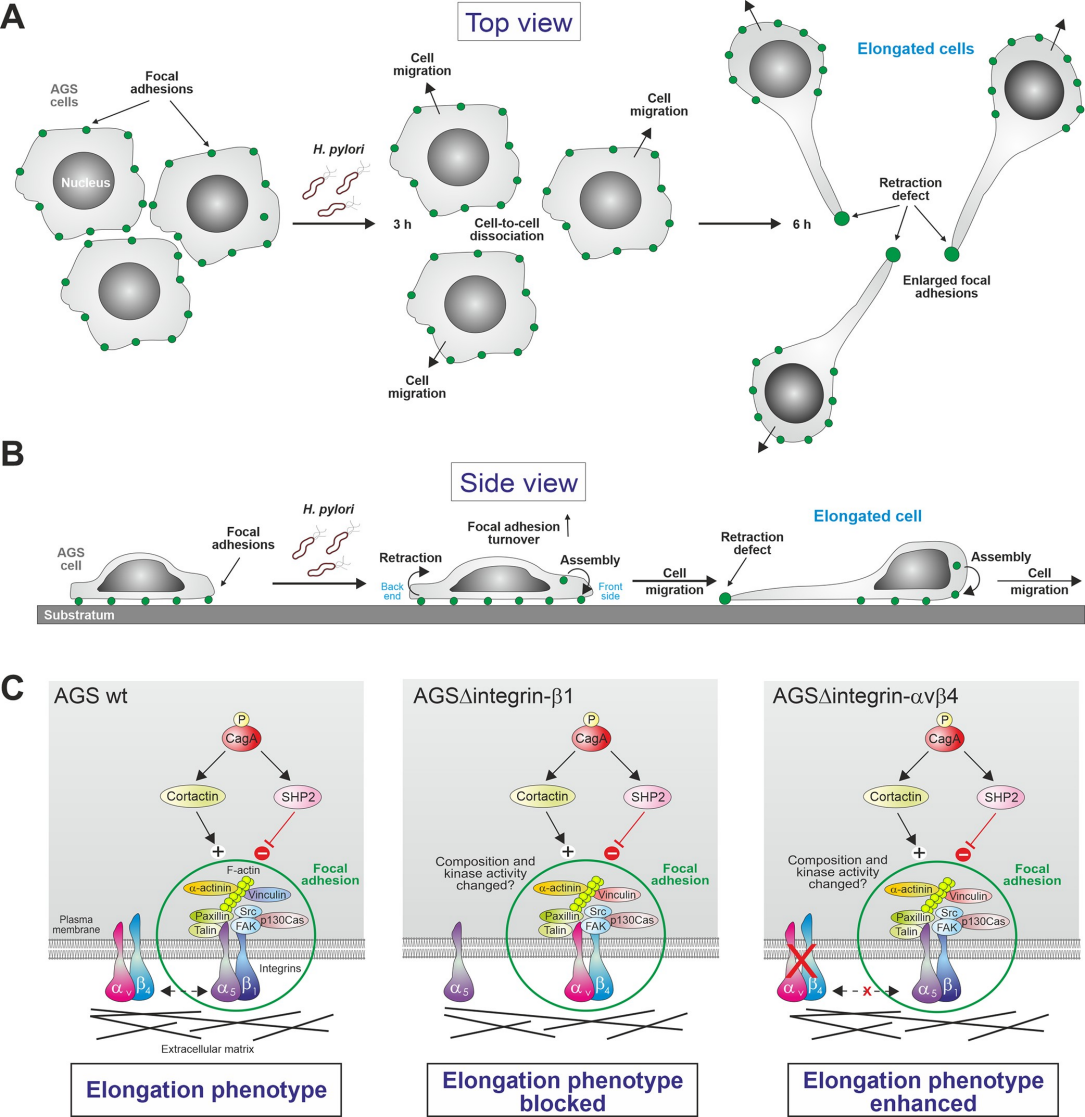
Schematic model for the elongation phenotype in *H*. *pylori* infected AGS cells and molecular events controlling phenotypic outcome. (A) Top view and (B) side view of infected AGS wild-type cells. Infection with T4SS-positive *H*. *pylori* induces host cell motility visible after 3–6 hours. The elongation phenotype depends on intracellular CagA^PY^ facilitating a cell retraction defect at the focal adhesions (FAs, green) during cell movement as indicated by arrows. Cell migration is characterized by the controlled FA assembly at the front side and their disassembly (retraction) at the back end of the cells as shown. The back ends become enlarged through the activities of CagA^PY^ and its disassembly is inhibited, resulting in elongated cell protrusions. (C) Proposed model for phenotypical outcome. The FA complexes comprise the indicated transmembrane integrin receptor heterodimers and a series of intracellular signaling factors including the tyrosine kinases Src and FAK and cytoskeletal proteins. Injected CagA^PY^ manipulates the activities of phosphatase SHP2 and the actin-binding protein cortactin, leading to the deregulation of FAK kinase activity. In this fashion, FAK controls cell adhesion to the extracellular matrix (bottom). A positive regulatory role is played by the integrin-α_5_β_1_ heterodimer to which some of the indicated factors directly interact, while ITGAvB4 knockout exhibits opposing effects. As a result, CagA^PY^ triggers the adhesion of wild-type AGS cells (left) resulting in the elongation phenotype, while this phenotype is blocked in ΔITGB1 cells (middle) and enhanced in ΔITGAvB4 cells (right). For more details, see text.

Third, what is the role of the high affinity T4SS-integrin-β_1_ interaction if not required for CagA delivery? It seems clear that *H*. *pylori* evolved at least four known T4SS proteins (CagA, CagI, CagL and CagY) that bind to the extracellular domain of integrin-β_1_ [1–3,6]. Zhao et al. [1] proposed that binding of these *cag* proteins to integrin-β_1_ heterodimers may allow tethering of the T4SS-pilus to the host cell resulting in a low level CagA translocation, but full CagA translocation requires the CEACAMs. In addition, the onset of intracellular signaling through integrins can be triggered. While there are no reports on the potential role of CagA, CagI and CagY for integrin signaling, a role of CagL has been described in detail. This includes the activation of transcription factor NF-κB leading to suppression of H,K-ATPase-α and gastric acid secretion [21], induction of IL-8 [22] and introduction of DNA double-strand breaks via endonucleases XPF and XPG leading to genome instability [23] as well as phosphorylation of cellular tyrosine kinases comprising Src, FAK, EGF receptor and its family member Her3/ErbB3 [24]. Remarkably, the ECM protein fibronectin can activate the same repertoire of kinases, except Her3/ErbB3 [20,25,26]. In addition, cultured host cells can robustly attach to immobilized CagL, which triggers FA formation and host cell spreading, a characteristic that was previously only known for ECM proteins like fibronectin [24]. Purified CagL could even complement the spreading defect of fibronectin knockout cells *in vitro*. These findings suggest that CagL displays functional mimicry with fibronectin [24]. In addition, CagL was previously shown to bind other integrins (AvB3, AvB5 and AvB6) with role in gastrin secretion and other functions [27–29]. Together, we propose that a direct binding of components of the T4SS such as CagL (and probably also CagA, CagI, and CagY) to integrin-β_1_ is to trigger intracellular signaling for bacterial advantage, but also enhance cell attachment and FA formation, thereby supporting the above discussed intracellular activities of CagA^PY^ towards FAK, with the overall goal to prevent excessive cell lifting during the course of infection. Future experiments should address these interactions of *H*. *pylori* with integrins in more detail and study the exact function of CEACAM receptors for CagA delivery.

## References

[1] Zhao Q, Busch B, Jiménez-Soto LF, Ishikawa-Ankerhold H, Massberg S, Terradot L, et al Integrin but not CEACAM receptors are dispensable for *Helicobacter pylori* CagA translocation. PLoS Pathog. 2018; 14: e1007359 10.1371/journal.ppat.1007359 30365569 PMC6231679

[2] Kwok T, Zabler D, Urman S, Rohde M, Hartig R, Wessler S, et al *Helicobacter* exploits integrin for type IV secretion and kinase activation. Nature 2007; 449: 862–866. 10.1038/nature06187 17943123

[3] Jiménez-Soto LF, Kutter S, Sewald X, Ertl C, Weiss E, Kapp U, et al *Helicobacter pylori* type IV secretion apparatus exploits beta1 integrin in a novel RGD-independent manner. PLoS Pathog. 2009; 5: e1000684 10.1371/journal.ppat.1000684 19997503 PMC2779590

[4] Javaheri A, Kruse T, Moonens K, Mejías-Luque R, Debraekeleer A, Asche CI, et al *Helicobacter pylori* adhesin HopQ engages in a virulence-enhancing interaction with human CEACAMs. Nat Microbiol. 2016; 2: 16189 10.1038/nmicrobiol.2016.189 27748768

[5] Königer V, Holsten L, Harrison U, Busch B, Loell E, Zhao Q, et al *Helicobacter pylori* exploits human CEACAMs via HopQ for adherence and translocation of CagA. Nat Microbiol. 2016; 2: 16188 10.1038/nmicrobiol.2016.188 27748756

[6] Kaplan-Türköz B, Jiménez-Soto LF, Dian C, Ertl C, Remaut H, Louche A, et al Structural insights into *Helicobacter pylori* oncoprotein CagA interaction with β1 integrin. Proc Natl Acad Sci U S A. 2012; 109: 14640–1465. 10.1073/pnas.1206098109 22908298 PMC3437852

[7] Segal ED, Cha J, Lo J, Falkow S, Tompkins LS. Altered states: involvement of phosphorylated CagA in the induction of host cellular growth changes by *Helicobacter pylori*. Proc Natl Acad Sci U S A. 1999; 96: 14559–14564. 10.1073/pnas.96.25.14559 10588744 PMC24475

[8] Backert S, Moese S, Selbach M, Brinkmann V, Meyer TF. Phosphorylation of tyrosine 972 of the *Helicobacter pylori* CagA protein is essential for induction of a scattering phenotype in gastric epithelial cells. Mol Microbiol. 2001; 42: 631–644. 10.1046/j.1365-2958.2001.02649.x 11722731

[9] Higashi H, Tsutsumi R, Muto S, Sugiyama T, Azuma T, Asaka M, et al SHP-2 tyrosine phosphatase as an intracellular target of *Helicobacter pylori* CagA protein. Science. 2002; 295: 683–686. 10.1126/science.1067147 11743164

[10] Bourzac KM, Botham CM, Guillemin K. *Helicobacter pylori* CagA induces AGS cell elongation through a cell retraction defect that is independent of Cdc42, Rac1, and Arp2/3. Infect Immun. 2007; 75: 1203–1213. 10.1128/IAI.01702-06 17194805 PMC1828586

[11] Tegtmeyer N, Wittelsberger R, Hartig R, Wessler S, Martinez-Quiles N, Backert S. Serine phosphorylation of cortactin controls focal adhesion kinase activity and cell scattering induced by *Helicobacter pylori*. Cell Host Microbe. 2011; 9: 520–531. 10.1016/j.chom.2011.05.007 21669400

[12] Selbach M, Moese S, Hurwitz R, Hauck CR, Meyer TF, Backert S. The *Helicobacter pylori* CagA protein induces cortactin dephosphorylation and actin rearrangement by c-Src inactivation. EMBO J. 2003; 22: 515–528. 10.1093/emboj/cdg050 12554652 PMC140734

[13] Tsutsumi R, Takahashi A, Azuma T, Higashi H, Hatakeyama M. Focal adhesion kinase is a substrate and downstream effector of SHP-2 complexed with *Helicobacter pylori* CagA. Mol Cell Biol. 2006; 26: 261–276. 10.1128/MCB.26.1.261-276.2006 16354697 PMC1317644

[14] Lawson CD, Burridge K. The on-off relationship of Rho and Rac during integrin-mediated adhesion and cell migration. Small GTPases. 2014; 5: e27958 10.4161/sgtp.27958 24607953 PMC4114617

[15] Shams H, Hoffman BD, Mofrad MRK. The "Stressful" Life of Cell Adhesion Molecules: On the Mechanosensitivity of Integrin Adhesome. J Biomech Eng. 2018; 140 10.1115/1.4038812 29272321

[16] Kleinschmidt EG, Schlaepfer DD. Focal Adhesion Kinase Signaling In Unexpected Places. Curr Opin Cell Biol. 2017; 45: 24–30. 10.1016/j.ceb.2017.01.003 28213315 PMC5482783

[17] LaFlamme SE, Akiyama SK, Yamada KM. Regulation of fibronectin receptor distribution. J Cell Biol. 1992; 117: 437–447. 10.1083/jcb.117.2.437 1373145 PMC2289425

[18] Akiyama SK, Yamada SS, Yamada KM, LaFlamme SE. Transmembrane signal transduction by integrin cytoplasmic domains expressed in single-subunit chimeras. J Biol Chem. 1994; 269: 15961–15964. 7515874

[19] Lukashev ME, Sheppard D, Pytela R. Disruption of integrin function and induction of tyrosine phosphorylation by the autonomously expressed beta 1 integrin cytoplasmic domain. J Biol Chem. 1994; 269: 18311–18314. 7518428

[20] Wennerberg K, Lohikangas L, Gullberg D, Pfaff M, Johansson S, Fässler R. Beta 1 integrin-dependent and -independent polymerization of fibronectin. J Cell Biol. 1996; 132: 227–238. 10.1083/jcb.132.1.227 8567726 PMC2120698

[21] Saha A, Backert S, Hammond CE, Gooz M, Smolka AJ. *Helicobacter pylori* CagL activates ADAM17 to induce repression of the gastric H, K-ATPase alpha subunit. Gastroenterology. 2010; 139: 239–248. 10.1053/j.gastro.2010.03.036 20303353 PMC2902712

[22] Gorrell RJ, Guan J, Xin Y, Tafreshi MA, Hutton ML, McGuckin MA, et al A novel NOD1- and CagA-independent pathway of interleukin-8 induction mediated by the *Helicobacter pylori* type IV secretion system. Cell Microbiol. 2013; 15: 554–570. 10.1111/cmi.12055 23107019

[23] Hartung ML, Gruber DC, Koch KN, Grüter L, Rehrauer H, Tegtmeyer N, et al *H*. *pylori-*Induced DNA Strand Breaks Are Introduced by Nucleotide Excision Repair Endonucleases and Promote NF-κB Target Gene Expression. Cell Rep. 2015; 13: 70–79. 10.1016/j.celrep.2015.08.074 26411687

[24] Tegtmeyer N, Hartig R, Delahay RM, Rohde M., Brandt S, Conradi J, et al A small fibronectin-mimicking protein from bacteria induces cell spreading and focal adhesion formation. J Biol Chem. 2010; 285: 23515–23526. 10.1074/jbc.M109.096214 20507990 PMC2906342

[25] Kuwada SK, Li X. Integrin alpha5/beta1 mediates fibronectin-dependent epithelial cell proliferation through epidermal growth factor receptor activation. Mol Biol Cell. 2000; 11: 2485–2496. 10.1091/mbc.11.7.2485 10888683 PMC14934

[26] Matsuo M, Sakurai H, Ueno Y, Ohtani O, Saiki I. Activation of MEK/ERK and PI3K/Akt pathways by fibronectin requires integrin alphav-mediated ADAM activity in hepatocellular carcinoma: a novel functional target for gefitinib. Cancer Sci. 2006; 97: 155–162. 10.1111/j.1349-7006.2006.00152.x 16441427 PMC11159791

[27] Buß M, Tegtmeyer N, Schnieder J, Dong X, Li J, Springer TA, Backert S, et al Specific high affinity interaction of *Helicobacter pylori* CagL with integrin α_V_ β_6_ promotes type IV secretion of CagA into human cells. FEBS J. 2019; 286: 3980–3997. 10.1111/febs.14962 31197920

[28] Conradi J, Huber S, Gaus K, Mertink F, Royo Gracia S, Strijowski U, et al Cyclic RGD peptides interfere with binding of the *Helicobacter pylori* protein CagL to integrins αVβ3 and α5β1. Amino Acids. 2012; 43: 219–232. 10.1007/s00726-011-1066-0 21915696

[29] Wiedemann T, Hofbaur S, Tegtmeyer N, Huber S, Sewald N, Wessler S, et al *Helicobacter pylori* CagL dependent induction of gastrin expression via a novel αvβ5-integrin-integrin linked kinase signalling complex. Gut. 2012; 61: 986–996. 10.1136/gutjnl-2011-300525 22287591

